# Objective evaluation of laparoscopic surgical skills in wet lab training based on motion analysis and machine learning

**DOI:** 10.1007/s00423-022-02505-9

**Published:** 2022-04-08

**Authors:** Koki Ebina, Takashige Abe, Kiyohiko Hotta, Madoka Higuchi, Jun Furumido, Naoya Iwahara, Masafumi Kon, Kou Miyaji, Sayaka Shibuya, Yan Lingbo, Shunsuke Komizunai, Yo Kurashima, Hiroshi Kikuchi, Ryuji Matsumoto, Takahiro Osawa, Sachiyo Murai, Teppei Tsujita, Kazuya Sase, Xiaoshuai Chen, Atsushi Konno, Nobuo Shinohara

**Affiliations:** 1 Graduate School of Information Science and Technology, Hokkaido University, Sapporo, Japan; 2 Department of Urology, Hokkaido University Graduate School of Medicine, North-15, West-7, North Ward, Sapporo, 060-8638 Japan; 3 Hokkaido University Clinical Simulation Center, Hokkaido University Graduate School of Medicine, Sapporo, Japan; 4 Department of Mechanical Engineering, National Defense Academy of Japan, Yokosuka, 239-8686 Japan; 5 Department of Mechanical Engineering and Intelligent Systems, Tohoku Gakuin University, Tagajo, 985-8537 Japan; 6 Graduate School of Science and Technology, Hirosaki University, Hirosaki, 036-8561 Japan

**Keywords:** Laparoscopic surgery, Simulation training, Motion capture, Machine learning, Surgical education

## Abstract

**Background:**

Our aim was to build a skill assessment system, providing objective feedback to trainees based on the motion metrics of laparoscopic surgical instruments.

**Methods:**

Participants performed tissue dissection around the aorta (tissue dissection task) and renal parenchymal closure (parenchymal-suturing task), using swine organs in a box trainer under a motion capture (Mocap) system. Two experts assessed the recorded movies, according to the formula of global operative assessment of laparoscopic skills (GOALS: score range, 5–25), and the mean scores were utilized as objective variables in the regression analyses. The correlations between mean GOALS scores and Mocap metrics were evaluated, and potential Mocap metrics with a Spearman’s rank correlation coefficient value exceeding 0.4 were selected for each GOALS item estimation. Four regression algorithms, support vector regression (SVR), principal component analysis (PCA)-SVR, ridge regression, and partial least squares regression, were utilized for automatic GOALS estimation. Model validation was conducted by nested and repeated k-fold cross validation, and the mean absolute error (MAE) was calculated to evaluate the accuracy of each regression model.

**Results:**

Forty-five urologic, 9 gastroenterological, and 3 gynecologic surgeons, 4 junior residents, and 9 medical students participated in the training. In both tasks, a positive correlation was observed between the speed-related parameters (e.g., velocity, velocity range, acceleration, jerk) and mean GOALS scores, with a negative correlation between the efficiency-related parameters (e.g., task time, path length, number of opening/closing operations) and mean GOALS scores. Among the 4 algorithms, SVR showed the highest accuracy in the tissue dissection task (${\mathrm{MAE}}_{\mathrm{median}}=2.2352$), and PCA-SVR in the parenchymal-suturing task (${\mathrm{MAE}}_{\mathrm{median}}=1.2714$), based on 100 iterations of the validation process of automatic GOALS estimation.

**Conclusion:**

We developed a machine learning–based GOALS scoring system in wet lab training, with an error of approximately 1–2 points for the total score, and motion metrics that were explainable to trainees. Our future challenges are the further improvement of onsite GOALS feedback, exploring the educational benefit of our model and building an efficient training program.

**Supplementary Information:**

The online version contains supplementary material available at 10.1007/s00423-022-02505-9.

## Introduction

Due to the widespread dissemination of minimally invasive surgery such as laparoscopic and robotic surgeries that require specific psychomotor skills, working hour restrictions, and ethical consideration regarding patient safety, simulation training outside the operating theater has been utilized in a broad range of surgical disciplines. In order to practice essential laparoscopic surgical skills, we also utilized simulation training using cadaveric porcine organs, including tissue dissection around the aorta, applying a Hem-o-lok in the vascular pedicle, and renal parenchymal closure, and reported its good construct validity [1]. Recently, we developed a novel motion capture (Mocap) system that could recognize each surgical instrument individually irrespective of instrument exchanges [2], and identified the motion characteristics of multiple surgical instruments according to the level of surgical experiences in a series of our aforementioned wet lab training. For example, in a task of tissue dissection around the swine aorta named “Task 1”, a shorter path length and faster velocity/acceleration/jerk were observed for both scissors and a Hem-o-lok applier in experts (> 50 laparoscopic surgeries), and especially in experts with > 100 cases, scissors moved more frequently in the close zone (0 < to < 2 cm from aorta) than in those with 50–99 cases [3].

Our goals are to characterize experts’ psychomotor skills based on motion metrics of surgical instruments, further improve surgical skills, and promote patient safety. As the next step, in order to enrich laparoscopic training programs, we aim to build a machine learning–based skill assessment system, in which the skill level is automatically evaluated and trainees receive explainable feedback based on the Mocap metrics of surgical instruments. In brief, two experts watched the recorded movies and evaluated the surgical dexterity according to the formula of global operative assessment of laparoscopic skills (GOALS) [4]. Using the mean GOALS scores as training data, we developed a machine learning–based GOALS scoring system in wet lab training, which could mitigate the educators’ workload, and promote self-training and peer-learning opportunities.

## Materials and methods

The institutional review board approved the present study (No. 018–0257). We previously reported the initial results based on the present Mocap system among urologic surgeons, a junior resident, and medical students (first data collection: *n* = 45, from December 2018 to February 2019) [3]. We continued data collection, including general and gynecologic surgeons (second data collection: from the end of May 2019 to September 2019). In the second data collection, participants performed tissue dissection around a swine aorta (task 1), and needle driving and making knots on kidney parenchyma (task 3), while only needle driving on kidney parenchyma (task 2) was not included because the characteristics of motion metrics divided by the level of surgical experiences were almost the same between tasks 2 and 3 in the first data collection [3]. Overall, a total of 70 participants performed 89 training sessions of tasks 1 and 3 during the whole study period (19 participants overlapped during the first and second data collections). Written informed consent was obtained regarding the use of their data for research.

We previously reported the details of the present training tasks [3]. Briefly, swine cadaveric organs were set in a box trainer (Endowork ProII®, Kyoto Kagaku, Japan). During the training, one of the 4 authors (TA, MH, JF, and NI) performed the role of a scopist, using a video system (VISERA Pro Video System Center OTV-S7Pro, Olympus, Japan). In task 1, participants were asked to dissect tissues around the aorta, dividing encountered mesenteric vessels after applying a Hem-o-lok clip. In task 3, using a 15-cm 2–0 CT-1 VICRYL® thread, participants were required to complete three square single-throw knots at 2 different sites on a kidney. If participants had trouble with the simulation, the scopist verbally guided each step of the task. All training sessions were video-recorded for later analyses. Demographic data and experience of laparoscopic surgeries were collected after the training session. The subjective mental workload was assessed by NASA Task Load Index after each training session for subsequent analysis.

### Motion capture analysis

**Fig. 1:**
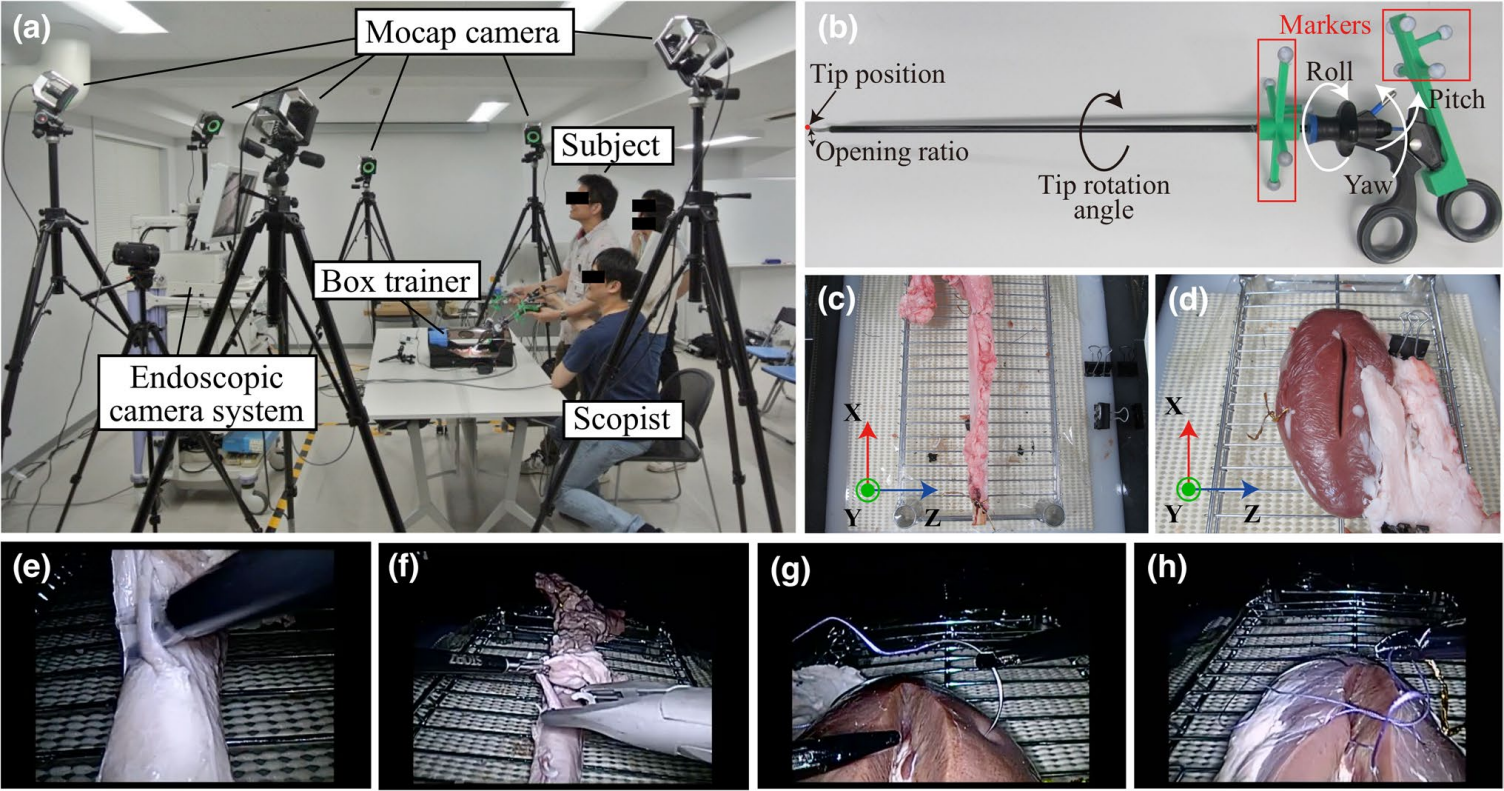
Photographs of the simulation training. **a** The Mocap system, which consisted of 6 infrared cameras (OptiTrack Prime 41, NaturalPoint Inc., USA), simultaneously tracked the movements of multiple surgical instruments during a series of training steps. **b** Scissors infrared reflective marker sets with an individual arrangement pattern were attached to handles of surgical instruments. **c** Swine aorta set in a dry box trainer. **d** Swine kidney set in a dry box trainer. **e** Task 1, a view of tissue dissection. **f** Task 1, a view of Hem-o-lok application. **g** Task 3, a view of needle driving. **h** Task 3, a view of making a knot

We previously reported the details of the present Mocap system [3]. Briefly, the Mocap system, which consists of 6 infrared cameras (OptiTrack Prime 41, NaturalPoint Inc., USA), simultaneously tracked multiple surgical instruments. Infrared reflective marker sets with a different arrangement pattern were attached to handles of each surgical device, which enabled our system to recognize each instrument individually regardless of the exchanges of instruments. The tip trajectories were calculated based on the positional relationship between the tip and handle. The track of the tip of a device (${x}_{i}, {y}_{i}, \mathrm{and} {z}_{i}$) was smoothed via the Savitzky-Golay filter [5], and its derivatives $\left(\frac{{d}^{j}{x}_{i}}{d{t}^{j}}, \frac{{d}^{j}{y}_{i}}{d{t}^{j}}, \mathrm{and} \frac{{d}^{j}{z}_{i}}{d{t}^{j}}\left(j=1\mathrm { to } 3\right)\right)$ were also calculated by the filter. The definitions of measurement outcomes (Mocap metrics) are summarized in Supplementary Table 1. In the second data collection, grasping forceps with strain gauges were utilized in task 1 in order to measure the grasping force and position of grasping forceps, although it was not a focus of the present study. Figure 1 shows pictures of the Mocap system, a surgical instrument with infrared markers, and endoscopic views of training tasks.

### Analyses and statistics

Two experts (TA and KH) evaluated the recorded movies in accordance with the GOALS formula [4]. Both raters performed more than 500 laparoscopic surgeries, and they were familiar with GOALS assessment because they were involved in the original validation study of the present wet lab model [1]. Anonymous video recordings were given to the two experts, and they independently scored the movies without any discussion or knowledge of participants’ backgrounds. The mean scores were utilized for subsequent analysis. The correlation was also evaluated by Spearman’s rank correlation coefficient. Next, correlations between mean GOALS scores and Mocap metrics were evaluated. For the subsequent machine learning analyses in order to develop an automatic skill assessment system according to the formula of GOALS, suitable Mocap metrics with a coefficient absolute value of more than 0.4 were selected for each GOALS item estimation based on the two authors’ discussions (KE and TA). Supplementary Table 2 summarizes the hypothesis for each item’s estimation, derived from the discussion process. In task 3, the score of “Tissue handling” was omitted because needle and thread manipulations were the main components of this drill.

In order to establish automatic GOALS assessment, we utilized the four regression algorithms: support vector regression (SVR), principal component analysis (PCA)-SVR, ridge regression (RR), and partial least squares regression (PLSR). Each algorithm has hyperparameters that must be determined before building a model. In this study, a grid search is performed to identify the best combination of parameters. The details of these algorithms and candidate parameters for the grid search are shown in Supplementary Table 3.

All Mocap metrics were normalized using robust *Z*-score normalization before inputting for these regression algorithms. The robust *Z* score, ${z}_{i},$ for data, ${x}_{i},$ can be calculated as follows:

$${z}_{i}=\frac{{x}_{i}-{x}_{\mathrm{m}}}{NIQR}.$$

Here, ${x}_{\mathrm{m}}$ is the median for data ${\varvec{x}}$, and $NIQR$ is the normalized interquartile range, calculated as $NIQR=0.7414\cdot IQR$ ($IQR$=interquartile range).

In order to evaluate the accuracies of each regression model, the mean absolute error (MAE) was used. MAE is calculated as follows:

$$\mathrm{MAE}=\frac{1}{n}\sum_{i=0}^{n}\left|{y}_{i}-\widehat{{y}_{i}}\right|,$$

where $n$ is the number of subjects, ${y}_{i}$ is an actual GOALS score of subject $i$, and $\widehat{{y}_{i}}$ is that predicted.

Model validation was conducted by nested and repeated k-fold cross-validation, a combined method of nested k-fold cross-validation and repeated cross-validation. Supplementary Fig. 1 shows the data flow of the validation process. In this validation method, nested k-fold cross-validation was repeated with different dataset divisions. The number of repetitions was set to 100 times in this study.

Spearman’s rank correlation coefficient was calculated using pandas ver. 1.1.4, data analysis library for Python (version 3.8.6.) [6]. All procedures of machine learning (parameter tuning, training, and validation models) were conducted using Scikit-learn, the machine learning library of Python [7]. The accuracies of each machine learning model calculated in the validation process were compared by the Kruskal–Wallis test. The Mann–Whitney *U* test was also utilized to assess the differences in model accuracy of each pair. Kruskal–Wallis and Mann–Whitney *U* tests were performed using JMP 14 (SAS, Japan).

## Results

Table 1 shows a summary of participants’ backgrounds. Forty-five urologic surgeons, 9 gastroenterological surgeons, 3 gynecologic surgeons, 4 junior residents, and 9 medical students voluntarily participated in the training during the study period. Previous experiences of laparoscopic surgery were as follows: 0–9: *n* = 20, 10–49: *n* = 18, 50–99: *n* = 7, 100–499: *n* = 18,  ≥ 500: *n* = 7. As described above, 19 joined the training multiple times, which resulted in a total of 89 training sessions. Due to video recording failure (task 1: *n* = 1 and task 3: *n* = 5), 88 movies of task 1 and 84 movies of task 3 were available for subsequent analyses.

**Table 1:** Summary of participants’ backgrounds

	*n* = 70
Age, years	Median 35 (range, 23–57)
Sex	Male/female = 61/9
Background	Urologic surgeon, *n* = 45
	Gastroenterological surgeon, *n* = 9
	Gynecologic surgeon, *n* = 3
	Junior resident, *n* = 4
	Medical student, *n* = 9
Experience of laparoscopic surgery	0–9, *n* = 20
	10–49, *n* = 18
	50–99, *n* = 7
	100–499, *n* = 18
	≥ 500, *n* = 7
Dominant hand	Right/left = 67/3

Supplementary Fig. 2 shows scatterplots of GOALS scores assessed by the two experts. Good interrater correlations of GOALS scores were confirmed in both tasks 1 and 3 (Spearman’s rank correlation coefficient: task 1 = 0.7773, task 3 = 0.878). As shown in Supplementary Table 4, a good correlation was also confirmed in each component (coefficient: 0.6662–0.8443).

**Fig. 2:**
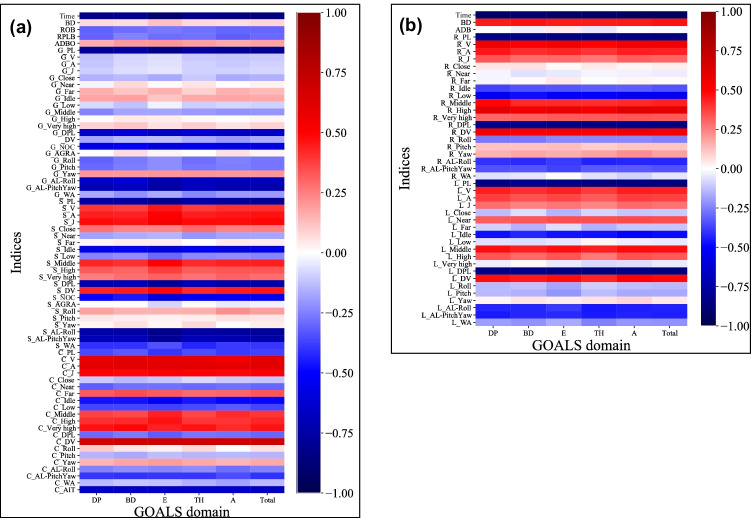
Heatmap of Spearman’s correlation coefficients between Mocap outcomes and mean GOALS scores (**a** task 1, **b** task 3). In both tasks, a positive correlation was observed between the speed-related parameters (e.g., velocity, velocity range, acceleration, jerk) and mean GOALS sores, with a negative correlation between the efficiency-related parameters (e.g., task time, path length, number of opening/closing operations) and the mean GOALS scores. “DP”, “BD”, “E”, “TH”, and “A” are items of GOALS. DP = Depth perception, BD = bimanual dexterity, E = efficiency, TH = tissue handling, A = autonomy. The prefixes of each item in the figure (“G”, “S”, “H”, “R”, “L”) are instruments. G = Grasping forceps, S = scissor forceps, C = clip applier, R = right needle holder, L = left needle holder. The suffixes of each item in the figure are Mocap parameters. Time = operative time, BD = bimanual dexterity, ROB = ratio of frequency of opening/closing for both forceps, RPLB = ratio of path length for both hands, ADBO = average distance between both forceps when opening/closing, ADB = average distance between both forceps, PL = path length, V = average velocity, A = average acceleration, J = average jerk, (Close, Near Far) = distribution of working area, (Idle, Low, Middle, High, Very high) = distribution of velocity, DPL = depth path length, DV = depth velocity, NOC = number of opening/closing operations, AGRA = average gripper rotation angle, (Roll, Pitch, Yaw) = average attitude angle, AL-Roll/AL-PitchYaw = angular length of Roll/Pitch and Yaw, WA = working area, AIT = average inserting time

Figure 2 (a: task 1, b: task 3) shows a heatmap of Spearman’s correlation coefficients between Mocap metrics and the mean GOALS scores (each item and total). In both tasks, a positive correlation was observed between the speed-related parameters (e.g., velocity, velocity range, acceleration, jerk) and mean GOALS scores, with a negative correlation between the efficiency-related parameters (e.g., task time, path length, number of opening/closing operations) and mean GOALS scores. In other words, Mocap analysis revealed that surgeons with high GOALS scores manipulated surgical instruments fast and dynamically, which resulted in greater efficiency.

Table 2 summarizes the correspondence between each GOALS item and the selected Mocap metrics with a coefficient of 0.4 or higher. For example, regarding depth perception in task 1, as summarized in Supplementary Table 2, because we hypothesized that metrics associated with movements along the sheath axis reflect depth perception, and "applying a Hem-o-lok clip on the vessel smoothly and quickly" strongly reflects a good spatial ability, we utilized the depth path length (DPL) and number of opening/closing operations for grasping forceps, DPL and depth velocity (DV) for scissor forceps, and all Mocap metrics with > 0.4 coefficient for the clip applier in subsequent machine learning analyses for the automatic skill assessment system.

**Table 2:** Correspondence table of each GOALS item and the selected Mocap parameters with a coefficient of more than 0.4

Task 1				
Item	General	Grasping forceps	Scissors	Clip applier
Depth perception		DPL, NOC	DPL, DV	V, A, J, Idle, High, Very high, DV, AIT
Bimanual dexterity	Time	PL, AL-Roll	PL, AL-Roll, AL-PitchYaw, WA	Idle, AL-PitchYaw, AIT
Efficiency	Time	PL, DPL, NOC, AL-Roll, AL-PitchYaw	PL, V, A, J, Idle, Middle, DPL, DV, NOC, AL-Roll, AL-PitchYaw, WA	V, A, J, Idle, High, Very high, DV, AIT
Tissue handling		PL, NOC, AL-Roll, AL-PitchYaw	PL, NOC, AL-Roll, AL-PitchYaw, WA	
Autonomy	Time		V, A, J, Idle, Middle	V, A, J, Idle, High, Very high, AIT
Task 3				
Item	General	Right needle holder	Left needle holder	
Depth perception		DPL, DV	DPL, AV	
Bimanual dexterity	Time, BD	PL, AL-Roll	PL, AL-Roll, AL-PitchYaw	
Efficiency	Time, BD	PL, V, A, Low, Middle, High, DPL, DV	PL, V, Idle, Middle, DPL, DV, AL-PitchYaw	
Tissue handling			Not calculated	
Autonomy	Time	V, A, Low, Middle, High	V, Idle, Middle	

*A* Average acceleration, *AIT* average inserting time, *AL-Roll/AL-PitchYaw* angular length of roll/pitch and yaw, *BD* bimanual dexterity, *DPL* depth path length, *DV* depth velocity, *(Idle, Low, Middle, High, Very high)* distribution of velocity, *J* average jerk, *NOC* number of opening/closing operations, *PL* path length, *V* average velocity, *WA* working area

**Fig. 3:**
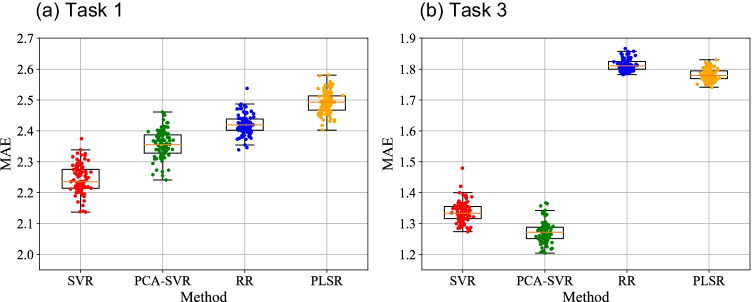
Box plots of mean absolute errors (MAEs) under repeated and nested cross-validation in each machine learning model in task 1 (**a**) and task 3 (**b**). SVR = support vector regression, PCA-SVR = principal component analysis-SVR, RR = ridge regression, and PLSR = partial least squares regression

**Fig. 4:**
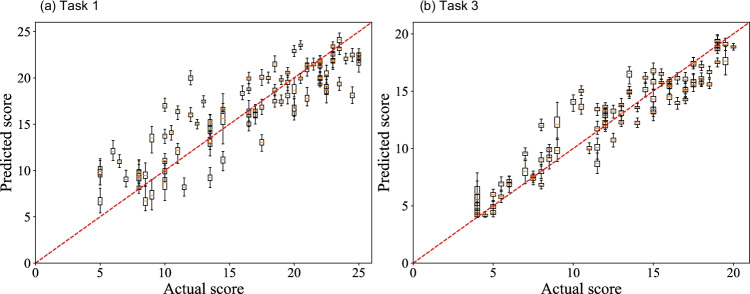
Box plots of actual vs. predicted GOALS scores of tasks 1 and 3. The predicted scores of task 1 were calculated by SVR, and those of task 3 were calculated by PCA-SVR. These models show the highest accuracy in each validation process. Since nested and repeated k-fold cross-validation was conducted in this study, the predicted GOALS score for one subject was obtained 100 times

Figure 3 shows box plots of MAEs regarding the accuracy of estimated GOALS scores (total) under repeated and nested cross-validation in each machine learning model (SVR, PCA-SVR, RR, and PLSR). Table 3 also shows a summary of the tests results including each GOALS component. Regarding the total GOALS score, the SVR method showed the highest accuracy in task 1 (Fig. 3a), and PCA-SVR in task 3 (Fig. 3b). The medians of the model accuracy of the 100 iterations of the validation process were ${\mathrm{MAE}}_{\mathrm{median}}=2.2352$ in task 1 (SVR), and ${\mathrm{MAE}}_{\mathrm{median}}=1.2714$ in task 3 (PCA-SVR). Figure 4 shows scatter plots of actual and predicted GOALS scores derived from 100 iterations. In task 1 (SVR), the errors between actual and predicted scores were smaller in the participants with actual scores > 15, and small in all participants in task 3 (PCA-SVR).

**Table 3:** Comparative summary of automatic GOALS assessment among the 4 regression algorithms

GOALS item	Median of MAEs (interquartile range)				*p* value						
	SVR	PCA-SVR	RR	PLSR	Kruskal–Wallis	MU test, SVR vs. PCA SVR	SVR vs. RR	SVR vs. PLSR	PCA-SVR vs. RR	PCA-SVR vs PLSR	RR vs. PLSR
Task 1											
Depth perception	0.5083 (0.4963–0.5221)	0.5509 (0.5363–0.5630)	0.5630 (0.5578–0.5684)	0.5912 (0.5807–0.6032)	< 0.0001	< 0.0001	< 0.0001	< 0.0001	< 0.0001	< 0.0001	< 0.0001
Bimanual dexterity	0.5234 (0.5116–0.5377)	0.5543 (0.5419–0.5673)	0.4964 (0.4925–0.5028)	0.5109 (0.5027–0.5208)	< 0.0001	< 0.0001	< 0.0001	< 0.0001	< 0.0001	< 0.0001	< 0.0001
Efficiency	0.4804 (0.4695–0.4943)	0.5102 (0.4951–0.5228)	0.4751 (0.4635–0.4871)	0.5261 (0.5166–0.5414)	< 0.0001	< 0.0001	0.0357	< 0.0001	< 0.0001	< 0.0001	< 0.0001
Tissue handing	0.6679 (0.6480–0.6816)	0.6114 (0.5971–0.6232)	0.6280 (0.6225–0.6371)	0.6408 (0.6310–0.6510)	< 0.0001	< 0.0001	< 0.0001	< 0.0001	< 0.0001	< 0.0001	< 0.0001
Autonomy	0.5323 (0.5195–0.5456)	0.5733 (0.5653–0.5861)	0.5803 (0.5681–0.5926)	0.6066 (0.5975–0.6133)	< 0.0001	< 0.0001	< 0.0001	< 0.0001	0.0259	< 0.0001	< 0.0001
Total	2.2352 (2.2141–2.2753)	2.3555 (2.3278–2.3868)	2.4917 (2.4020–2.4385)	2.4933 (2.4673–2.5134)	< 0.0001	< 0.0001	< 0.0001	< 0.0001	< 0.0001	< 0.0001	< 0.0001
Task 3											
Depth perception	0.3671 (0.3611–0.3740)	0.3575 (0.3512–0.3631)	0.4147 (0.4112–0.4184)	0.4077 (0.4053–0.4110)	< 0.0001	< 0.0001	< 0.0001	< 0.0001	< 0.0001	< 0.0001	< 0.0001
Bimanual dexterity	0.4430 (0.4318–0.4559)	0.4575 (0.4435–0.4729)	0.5318 (0.5265–0.5376)	0.5152 (0.5096–0.5225)	< 0.0001	< 0.0001	< 0.0001	< 0.0001	< 0.0001	< 0.0001	< 0.0001
Efficiency	0.3843 (0.3748–0.4004)	0.3642 (0.3546–0.3791)	0.5127 (0.5042–0.5230)	0.5203 (0.5136–0.5303)	< 0.0001	< 0.0001	< 0.0001	< 0.0001	< 0.0001	< 0.0001	< 0.0001
Autonomy	0.3853 (0.3758–0.3944)	0.3696 (0.3620–0.3830)	0.5326 (0.5279–0.5386)	0.5153 (0.5103–0.5209)	< 0.0001	< 0.0001	< 0.0001	< 0.0001	< 0.0001	< 0.0001	< 0.0001
Total	1.3336 (1.3156–1.3554)	1.2714 (1.2513–1.2879)	1.8105 (1.8000–1.8248)	1.7794 (1.7693–1.7945)	< 0.0001	< 0.0001	< 0.0001	< 0.0001	< 0.0001	< 0.0001	< 0.0001

## Discussion

Using motion metrics of the instruments, several previous studies were aimed to measure surgical skills [8, 9], and machine learning showed good performance to classify laparoscopic surgeons according to previous surgical experience [10–13]. For example, Oropesa et al. compared three methods: linear discriminant analysis (LDA), SVM, and an adaptive neuro-fuzzy inference system (AN-FIS), to classify 42 participants according to previous surgical experience (> 10 laparoscopic surgeries performed vs. < 10) by leave-one-out cross-validation [12]. Instrument movements were captured in three box trainer tasks (peg grasping task, task that requires placing three elastic bands through their corresponding posts, and coordinated peg transfer task) by the TrEndo tracking system [10]. They observed that the mean accuracy of classification was 71% with LDA, 78.2% with SVM, and 71.7% with AN-FIS. Overall, previous researchers utilized simple training tasks such as “peg transfer”, “pattern cutting”, or “suturing”, which did not require the exchange of surgical instruments, utilized previous surgical experience as a surrogate marker of surgical competence, and focused on the differential accuracy of each machine learning model regarding surgical competency, not providing comprehensive feedback to trainees based on the movements of surgical instruments. Rather, in the present study, using relatively complex training tasks (task 1: tissue dissection around a swine aorta, task 3: renal parenchymal closure) for which we previously reported good construct validity [1], we aimed to develop a wet-lab training model that offered completely objective feedback to trainees according to the GOALS formula.

Because our prior study showed that the level of surgical experience was not always associated with surgical dexterity on Mocap-based evaluation, we reviewed the recorded movies, and scored those according to the GOALS formula. GOALS is an already validated and widely used assessment tool for grading laparoscopic surgical skills, and consists of five items: depth perception, bimanual dexterity, efficiency, tissue handling, and autonomy [4]. Each item is scored on a Likert scale between 1 and 5, which results in a total score between 5 and 25. As a result, we observed good interrater correlation between the two experts for the total score and for each item both in tasks 1 and 3 (Supplementary Fig. 2, and Supplementary Table 4), and the mean scores were utilized for subsequent analyses.

As the next step, we evaluated the correlation between the mean GOALS scores and Mocap metrics. In both tasks, a positive correlation was observed in the speed-related metrics (e.g., velocity, velocity range, acceleration, jerk), and a negative correlation in the efficiency-related metrics (e.g., task time, path length, number of opening/closing operations). Because we aimed to calculate each GOALS item’s score according to its original meaning, potential Mocap metrics were selected based on the two authors’ discussions, as summarized in Supplementary Table 2. For example, as described above, we utilized the depth path length (DPL) and number of opening/closing operations with grasping forceps (we hypothesized that poor depth perception results in failure to grasp tissues), DPL and depth velocity (DV) with scissor forceps, and all Mocap metrics with > 0.4 coefficient with the clip applier regarding depth perception in task 1. Because we considered that “applying a Hem-o-lok clip on a pedicle smoothly” strongly reflected the skill of depth perception, all Mocap metrics with > 0.4 coefficient with the clip applier were included. In task 3, after discussion, we discontinued “Tissue handling” calculation because the task consisted of needle and thread control, not tissue manipulation.

In the present study, we utilized the four major algorithms for automatic GOALS estimation. As SVR and PCA-SVR are non-linear regression methods, and RR and PLSR are linear regression methods, we aimed to utilize a variety of regression methods. Regarding the validation process, in general, “leave-one-out cross-validation” has a tendency whereby the generalization error often has high variance because the model is constructed by [*n*-1] samples out of a total of [*n*] samples, and the constructed models may be identical to each other and the same as models built from the entire training set, which may result in overfitting. For these reasons, we performed k-fold cross-validation. In addition, in order to optimize hyper parameters of a model while avoiding overfitting to the dataset, we utilized nested cross-validation by nesting k-fold cross-validation. Regarding the accuracy of the machine learning model, in general, MAE and root mean squared error (RMSE) are frequently utilized. Regarding RMSE, because it uses the square error for calculation, it might be strongly influenced by outliers. In the present study, aiming to evaluate the error equally among all data, we used MAE, which utilizes an absolute error value for accuracy estimation.

As presented in Fig. 3 and Table 3, the SVR method showed the highest accuracy in task 1, and PCA-SVR in task 3. The medians of the model accuracy of 100 iterations of the validation process were ${\mathrm{MAE}}_{\mathrm{median}}=2.2352\mathrm{ points}$ in task 1 (SVR), and ${\mathrm{MAE}}_{\mathrm{median}}=1.2714\mathrm{ points}$ in task 3 (PCA-SVR). In other words, the SVR model can estimate the GOALS score with an error of approximately 2.2 points in the range of GOALS scores of 5–25 in task 1, and PCA-SVR with an error of approximately 1.3 points in the range of GOALS scores of 4–20 in task 3. As shown in Fig. 4, the errors between actual and predicted scores were smaller in the participants with actual scores > 15 in task 1, and small in all participants in task 3 (PCA-SVR). Our observations suggest that in task 1, automatic feedback is more reliable in better surgeons, while reliable in a range of surgeons in task 3, and both tasks should be included in training drills in order to provide accurate feedback to participants according to the GOALS formula. Our future challenge is further improvement for onsite GOALS feedback. A randomized control study, with the hypothesis that motion capture–based feedback enhances the initial phase of the learning curve, would be an interesting study to address the educational benefit of the current model. In addition, we consider that our automated GOALS score can be used as part of a skill credentialing system.

Limitations of this study include the small sample size, lack of qualitative assessment of the tissue dissection and intracorporeal knot suture themselves, and heterogeneity including, for example, three surgeons were left-handed, although they performed actual surgeries with a right-handed style. Regarding the accuracy of the tip position, we previously compared the tip position calculated by the positional relationship between the tip and handle, with the actual tip position, which was measured by tracking the marker attached to the tip of devices [2]. The positional errors were less than 2 mm, and the current smoothing process also might influence the metrics, although we consider the errors to be within an acceptable range.

In addition, the present study was never free from the uncertainty derived from human assessment of the GOALS score, although we utilized mean scores from two experts, who independently assessed the movies in a blind manner in order to mitigate human bias. The selection process of Mocap metrics for each GOALS item calculation was also not free from bias. As described above, in order to mitigate the risk of overfitting, we utilized a combined method of nested and repeated k-fold cross-validation. In the cross validation process, training sets were not used as test sets in each iteration. We consider that this method can mitigate the risk of overfitting compared with “leave-one-out cross-validation. However, the data splitting method (training and validation) is not free from overfitting or model selection bias, and we need to validate our model with an external cohort. In order to validate our system, we just started new data collection, with several improvements including the use of another motion camera system with ease of portability that does not require calibration (OptiTrack V120: Trio, NaturalPoint Inc., USA). Using this system, we are now collecting Mocap data in cadaveric simulation trainings (laparoscopic nephrectomy task). Because it is a more complicated and long-duration task, we might be able to analyze the change of Mocap characteristics according to the progress of the task, with the hypothesis that experts have specific Mocap features in terms of “robustness” to long surgery. Furthermore, to clarify tacit knowledge regarding surgical dexterity, different approaches such as explainable artificial intelligence might be necessary. Nevertheless, the machine learning algorithms automatically enabled us to asses surgical psychomotor skills based on the motion metrics of surgical instruments.

## Conclusions

Using machine learning algorithms, we developed a Mocap-based skill assessment system in wet lab training, with a total GOALS score error of approximately 1–2 points, which can provide completely objective feedback to trainees. Our future challenges are the further improvement of onsite GOALS feedback, exploring the educational benefit of our model, and building an efficient training program.

## Supplementary Information

Below is the link to the electronic supplementary material.

ESM 1Supplementary Figure 1. Data flow of validation process, Nested k-fold cross-validation consists of two validation processes, an outer cross-validation (Outer CV) and inner cross-validation (Inner CV). In this study, each cross-validation process was conducted by 10-fold cross-validation. In the outer CV, the dataset was divided into 10 groups; 9 groups were used as a training set, and 1 group was used as a testing set. The model accuracy was evaluated by repeating the dividing process 10 times so that all groups were evaluated as the testing set. The inner CV was conducted using training data of the outer CV. The input dataset was divided into 10 groups; 9 groups were used as the training set, and 1 group was used as a validation set. The model accuracy was calculated in the same way as the outer CV. The grid search for hyper parameter tuning was conducted in the inner CV. The model accuracy for all combinations of the candidate parameter was compared. The best parameter showing the highest accuracy of all candidate parameters was used to build the model of the outer CV. In this study, stratified cross-validation was used in the outer CV. The dataset was divided into 4 groups according to the 1-3 quartiles of GOALS scores, and data from each group were divided into 10 groups of 10-fold cross-validation so that there was no bias. The inner CV was conducted as normal 10-fold cross-validation due to the limitation of machine learning libraries used in this study. Because the predicted GOALS scores calculated in the outer CV might be outside the original GOALS score range (1-5), the predicted scores, $\hat{y},$ in the outer CV were modified with the following formula: $${\hat{y}}_{\mathrm{mod}}=\left\{\begin{array}{c}5\left(\hat{y}>5\right)\\ {}1\left(\hat{y}<1\right)\end{array}\right.$$. The overall model accuracy in the validation process was calculated using the modified score ${\hat{y}}_{\mathrm{mod}}$. (PPTX 71 kb)

ESM 2 Supplementary Figure 2. Scatterplots of GOALS scores assessed by the two experts. Good interrater correlation of GOALS scores was confirmed in both Tasks 1 and 3 (Spearman’s rank correlation coefficient: Task 1=0.7773, Task 3=0.878). (PPTX 103 kb)

Supplementary file3 (DOCX 31 KB)

Supplementary file4 (DOCX 917 KB)

Supplementary file5 (DOCX 14 KB)

Supplementary file6 (DOCX 64 KB)

## Declarations

The institutional review board approved the present study (No. 018–0257).
