# Supplementary figures and images for: Objective evaluation of laparoscopic surgical skills in wet lab training based on motion analysis and machine learning
Source: Langenbecks Arch Surg. 2022 Apr 8;407(5):2123–32. doi: 10.1007/s00423-022-02505-9 (PMC9399206; doi:10.1007/s00423-022-02505-9)

## Slide 1
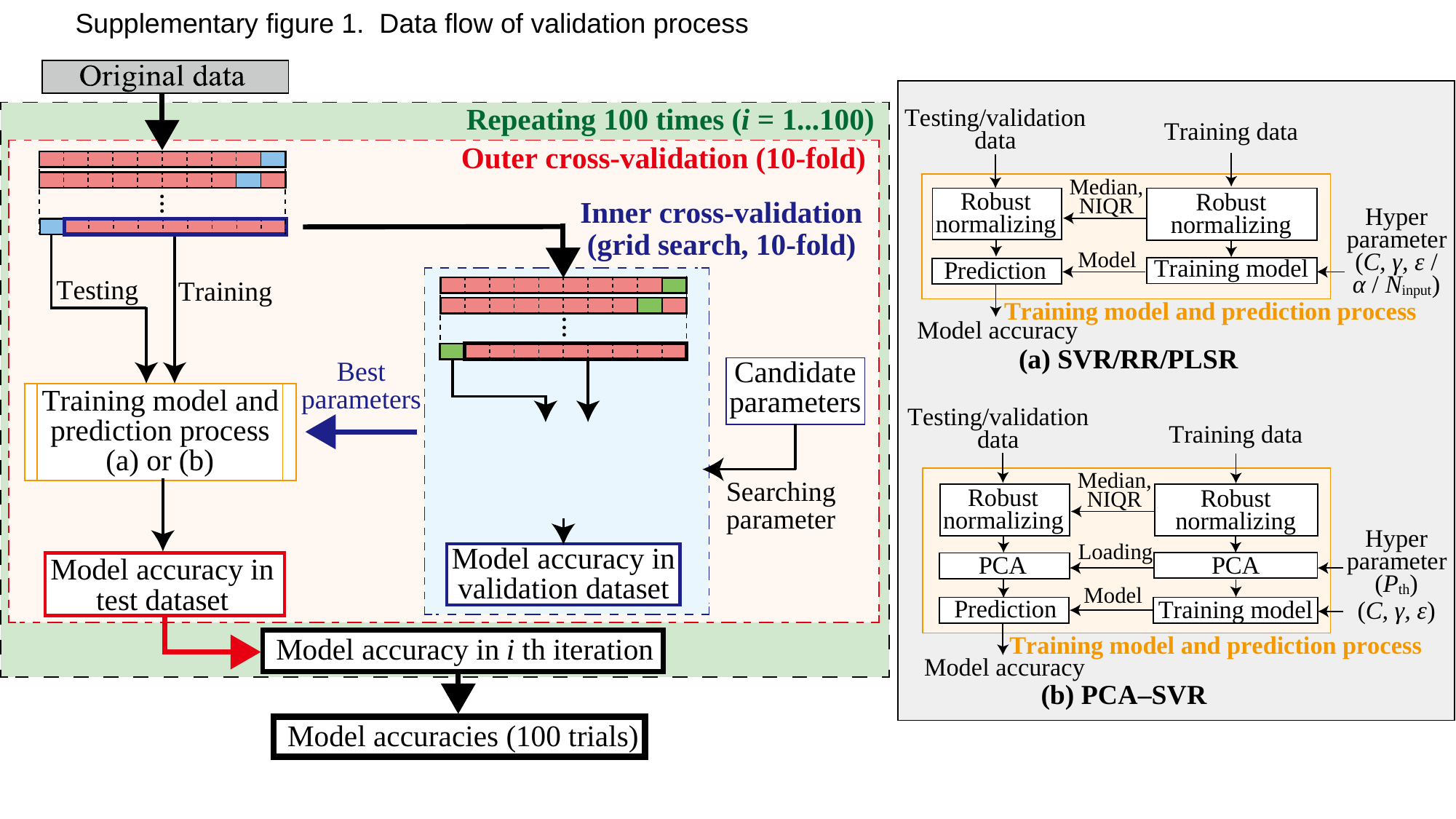

Supplementary figure 1. Data flow of validation process

Supplement: Supplementary file 1 — Supplementary Figure 1. Data flow of validation process, Nested k-fold cross-validation consists of two validation processes, an outer cross-validation (Outer CV) and inner cross-validation (Inner CV). In this study, each cross-validation process was conducted by 10-fold cross-validation. In the outer CV, the dataset was divided into 10 groups; 9 groups were used as a training set, and 1 group was used as a testing set. The model accuracy was evaluated by repeating the dividing process 10 times so that all groups were evaluated as the testing set. The inner CV was conducted using training data of the outer CV. The input dataset was divided into 10 groups; 9 groups were used as the training set, and 1 group was used as a validation set. The model accuracy was calculated in the same way as the outer CV. The grid search for hyper parameter tuning was conducted in the inner CV. The model accuracy for all combinations of the candidate parameter was compared. The best parameter showing the highest accuracy of all candidate parameters was used to build the model of the outer CV. In this study, stratified cross-validation was used in the outer CV. The dataset was divided into 4 groups according to the 1-3 quartiles of GOALS scores, and data from each group were divided into 10 groups of 10-fold cross-validation so that there was no bias. The inner CV was conducted as normal 10-fold cross-validation due to the limitation of machine learning libraries used in this study. Because the predicted GOALS scores calculated in the outer CV might be outside the original GOALS score range (1-5), the predicted scores, $\hat{y},$y^, in the outer CV were modified with the following formula: [file 423_2022_2505_MOESM1_ESM.pptx]
