# Supplementary material for: Objective evaluation of laparoscopic surgical skills in wet lab training based on motion analysis and machine learning
Source: Langenbecks Arch Surg. 2022 Apr 8;407(5):2123–32. doi: 10.1007/s00423-022-02505-9 (PMC9399206; doi:10.1007/s00423-022-02505-9)
Supplement: Supplementary file 2 — Supplementary Figure 2. Scatterplots of GOALS scores assessed by the two experts. Good interrater correlation of GOALS scores was confirmed in both Tasks 1 and 3 (Spearman’s rank correlation coefficient: Task 1=0.7773, Task 3=0.878). (PPTX 103 kb) [file 423_2022_2505_MOESM2_ESM.pptx]

## Slide 1
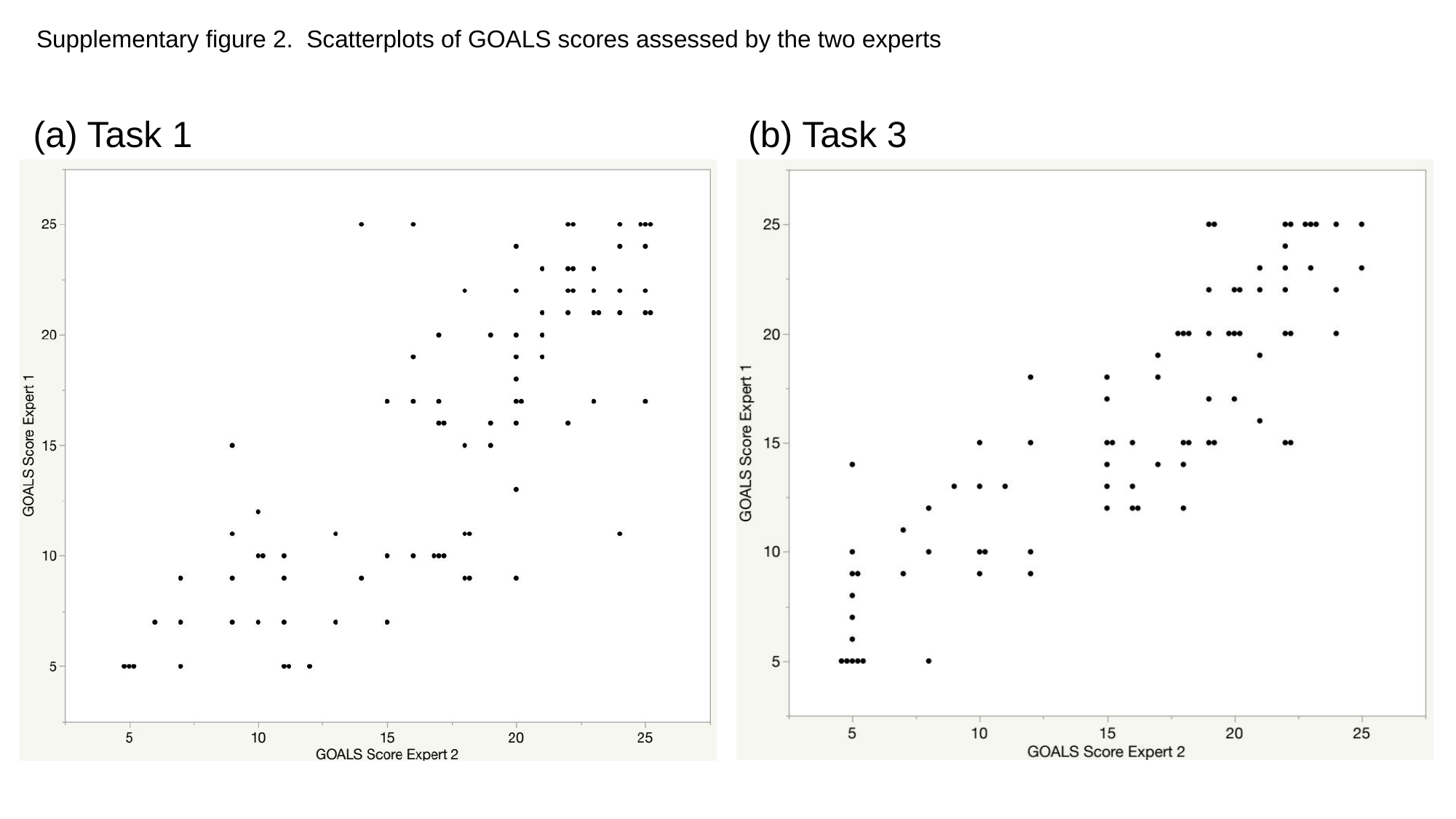

Supplementary figure 2. Scatterplots of GOALS scores assessed by the two experts
(a) Task 1
(b) Task 3
