# Supplementary material for: Objective evaluation of laparoscopic surgical skills in wet lab training based on motion analysis and machine learning
Source: Langenbecks Arch Surg. 2022 Apr 8;407(5):2123–32. doi: 10.1007/s00423-022-02505-9 (PMC9399206; doi:10.1007/s00423-022-02505-9)
Supplement: Supplementary file 3 — Supplementary file3 (DOCX 31 KB) [file 423_2022_2505_MOESM3_ESM.docx]

Supplementary Table 1. The definitions of measurement outcomes

| Indices | | Unit | Definitions | Formulae |
| --- | --- | --- | --- | --- |
| General | |  |  |  |
| Operative time (Time) | | (s) | Total time to complete a task. |  |
| Bimanual dexterity (BD) | |  | This is an index for evaluating the coordinated movement between both hands, and is derived from the relationship between the tip velocities of instruments controlled by both hands at the same time [10]. | $\frac{\sum_{i=1}^{n} \left( v_{\mathrm{left}}\left( i \right)- V_{\mathrm{left}} \right)\left( v_{\mathrm{right}}\left( i \right)- V_{\mathrm{right}} \right)}{\sqrt{\sum_{i=1}^{n} \left( v_{\mathrm{left}}\left( i \right)- V_{\mathrm{left}} \right)^{2}\sum_{i=1}^{n} \left( v_{\mathrm{right}}\left( i \right)- V_{\mathrm{right}} \right)^{2}}}$ ,  Where $n$ is the total number of Mocap frames of the measured task, and $v_{\mathrm{left}}\left( i \right)$ and $v_{\mathrm{right}}\left( i \right)$ are tip velocities of instruments controlled by left and right hands in frame $i$, respectively. These velocities are calculated as follows: $v_{\mathrm{left}}(i)=\left\Vert\boldsymbol{v}_{\mathbf{left}}\left( i \right) \right\Vert, v_{\mathrm{right}}(i)=\left\Vert\boldsymbol{v}_{\mathbf{right}}\left( i \right) \right\Vert$. Here,$V_{\mathrm{left}}$ and $V_{\mathrm{right}}$ are average tip velocities of instruments controlled by left and right hands, respectively. |
| Ratio of frequency of opening/closing both instruments (ROB) | |  | Ratio of frequency of opening/closing of instruments controlled by left and right hands in the dissection task. (Left hand: grasping forceps, right hand: scissors) | $\frac{\mathrm{NOC}_{\mathrm{grasper}}}{\mathrm{NOC}_{\mathrm{scissor}}}$, where $\mathrm{NOC}_{\mathrm{grasper}}$ is the frequency of opening/closing grasping forceps, and $\mathrm{NOC}_{\mathrm{scissor}}$ is that of scissors. |
| Ratio of path length for both hands (RPLB) | |  | Ratio of path length of the tip position in both hands in the dissection task. (Left hand: grasping forceps, right hand: scissors) | $\frac{\mathrm{PL}_{\mathrm{grasper}}}{\mathrm{PL}_{\mathrm{scissor}}}$, where $\mathrm{PL}_{\mathrm{grasper}}$ is the path length of grasping forceps, and $\mathrm{PL}_{\mathrm{scissor}}$ is that of scissors. |
| Average distance between both instruments when opening/closing (ADBO) | | (m) | Average distance between tip positions of both instruments when opening/closing grasping forceps in the dissection task. | $\frac{\sum_{i=1}^{\mathrm{NOC}_{\mathrm{grasper}}} \left\Vert\boldsymbol{p}_{\mathbf{tip}\left( \boldsymbol{grasper\_oc} \right)}(i)-\boldsymbol{p}_{\mathbf{tip}\left( \boldsymbol{scissor\_oc} \right)}\mathbf{(}i\mathbf{)} \right\Vert}{\mathrm{NOC}_{\mathrm{grasper}}}$,  where $\boldsymbol{p}_{\mathbf{tip}\left( \boldsymbol{grasper\_OC} \right)}\left( i \right)$ is the tip position of grasping forceps in the $i$ th opening/closing operation, and $\boldsymbol{p}_{\mathbf{tip}\left( \boldsymbol{scissor\_oc} \right)}\left( i \right)$ is that of scissors. |
| Average distance between both instruments (ADB) | | (m) | Average distance between tip positions of both instruments in the suturing/knotting task. | $\frac{\sum_{i=1}^{n} \left\Vert\boldsymbol{p}_{\mathbf{tip}\left( \mathbf{right} \right)}(i)-\boldsymbol{p}_{\mathbf{tip}\left( \mathbf{left} \right)}\mathbf{(}i\mathbf{)} \right\Vert}{n}$,  where $\boldsymbol{p}_{\mathbf{tip}\left( \mathbf{left} \right)}\mathbf{(}i\mathbf{)}$ is the tip position of the left needle holder in frame $i$, and $\boldsymbol{p}_{\mathbf{tip}\left( \mathbf{right} \right)}\mathbf{(}i\mathbf{)}$ is that of the right needle holder. |
| Instrument motion | |  |  |  |
| Path length (PL) | | (m) | Total length of the tip trajectory of an instrument. Trajectory that lies outside the box trainer is excluded from the calculation. | $\sum_{i=1}^{n-1} \left\Vert\boldsymbol{p}_{\mathbf{tip}}\left( i+1 \right)-\boldsymbol{p}_{\mathbf{tip}}\left( i \right) \right\Vert$,  where, $\boldsymbol{p}_{\mathbf{tip}}\left( i \right)$ is the tip position of an instrument in frame $i$. The positional vector $\boldsymbol{p}_{\mathbf{tip}}$ is smoothed by the Savitzky-Golay filter. |
| Average velocity (V) | | $\left( \mathrm{cm}/s \right)$ | Average velocity of the tip of an instrument. | $\frac{1}{n}\sum_{i=1}^{n} \left\Vert\frac{d}{dt}\boldsymbol{p}_{\mathrm{tip}}(i) \right\Vert$, where $\frac{d}{dt}\boldsymbol{p}_{\mathrm{tip}}(i)$ is directly obtained by the Savitzky-Golay filter. |
| Average acceleration (A) | | $\left( \mathrm{cm}/{s^{2}} \right)$ | Average acceleration of the tip of an instrument. | $\frac{1}{n}\sum_{i=1}^{n} \left\Vert\frac{d^{2}}{dt^{2}}\boldsymbol{p}_{\mathrm{tip}}(i) \right\Vert$, where $\frac{d^{2}}{dt^{2}}\boldsymbol{p}_{\mathrm{tip}}(i)$ is directly obtained by the Savitzky-Golay filter. |
| Average jerk (J) | | $\left( \mathrm{cm}/{s^{3}} \right)$ | Average jerk of the tip of an instrument. Jerk is the changing rate of acceleration. | $\frac{1}{n}\sum_{i=1}^{n} \left\Vert\frac{d^{3}}{dt^{3}}\boldsymbol{p}_{\mathrm{tip}}(i) \right\Vert$, where $\frac{d^{3}}{dt^{3}}\boldsymbol{p}_{\mathrm{tip}}(i)$ is directly obtained by the Savitzky-Golay filter. |
| Distribution of working area　(Close/Near/Far zone) | Close | (%) | The ratio of the path length that is moved around the target object in a certain area to the total path length. In the dissection task, the target object was designated by placing both instruments on the start/end point of the dissecting area of the aorta for 5 s. The target object in the suturing/knotting task was designated in the same way on the incised line of the kidney parenchyma. | $\frac{\sum_{i=1}^{n-1} \left\Vert\boldsymbol{p}_{\mathrm{tip}}\left( i+1 \right)-\boldsymbol{p}_{\mathrm{tip}}\left( i \right) \right\Vert}{\mathrm{PL}}$ $=\left\{ i\in\left( 0,\ldots n-1 \right)\vert\left\Vert\left( \boldsymbol{p}_{\mathbf{start}}+l\frac{\boldsymbol{p}_{\mathbf{end}}\boldsymbol{-}\boldsymbol{p}_{\mathbf{start}}}{\left\Vert\boldsymbol{p}_{\mathbf{end}}\boldsymbol{-}\boldsymbol{p}_{\mathbf{start}} \right\Vert} \right)-\boldsymbol{p}_{\mathbf{tip}}\left( i+1 \right) \right\Vert<0.02 \right\} \left( \mathrm{cm} \right)$, where $\boldsymbol{p}_{\mathbf{start}}$ and $\boldsymbol{p}_{\mathbf{end}}$ are positions of start and end points of the target object, and $l$ is defined as follows: $l=\left\{ \begin{aligned} 0 \\ d \\ \left\Vert\boldsymbol{p}_{\mathbf{end}}\boldsymbol{-}\boldsymbol{p}_{\mathbf{start}} \right\Vert\end{aligned} \right.\begin{matrix} \left( d\leq0 \right) \\ \left( 0<d<\left\Vert\boldsymbol{p}_{\mathbf{end}}\boldsymbol{-}\boldsymbol{p}_{\mathbf{start}} \right\Vert\right) \\ \left( \left\Vert\boldsymbol{p}_{\mathbf{end}}\boldsymbol{-}\boldsymbol{p}_{\mathbf{start}} \right\Vert\leq d \right) \end{matrix} .$  Here, $d$ is calculated as follows: $d=\frac{\frac{\boldsymbol{p}_{\mathbf{end}}\boldsymbol{-}\boldsymbol{p}_{\mathbf{start}}}{\left\Vert\boldsymbol{p}_{\mathbf{end}}\boldsymbol{-}\boldsymbol{p}_{\mathbf{start}} \right\Vert}\boldsymbol{\cdot}\left( \boldsymbol{p}_{\mathbf{tip}}\left( i+1 \right)-\boldsymbol{p}_{\mathbf{start}} \right)}{\left\Vert\boldsymbol{p}_{\mathbf{end}}\boldsymbol{-}\boldsymbol{p}_{\mathbf{start}} \right\Vert^{2}}$. |
|  | Near |  |  | $\frac{\sum_{i=1}^{n-1} \left\Vert\boldsymbol{p}_{\mathbf{tip}}\left( i+1 \right)-\boldsymbol{p}_{\mathbf{tip}}\left( i \right) \right\Vert}{PL}$ $=\left\{ i\in\left( 0,\ldots n-1 \right)\vert0.02\leq\left\Vert\left( \boldsymbol{p}_{\mathbf{start}}+l\frac{\boldsymbol{p}_{\mathbf{end}}\boldsymbol{-}\boldsymbol{p}_{\mathbf{start}}}{\left\Vert\boldsymbol{p}_{\mathbf{end}}\boldsymbol{-}\boldsymbol{p}_{\mathbf{start}} \right\Vert} \right)-\boldsymbol{p}_{\mathbf{tip}}\left( i+1 \right) \right\Vert<0.04 \right\} \left( \mathrm{cm} \right).$ |
|  | Far |  |  | $\frac{\sum_{i=1}^{n-1} \left\Vert\boldsymbol{p}_{\mathbf{tip}}\left( i+1 \right)-\boldsymbol{p}_{\mathbf{tip}}\left( i \right) \right\Vert}{PL}$ $=\left\{ i\in\left( 0,\ldots n-1 \right)\vert0.04\leq\left\Vert\left( \boldsymbol{p}_{\mathbf{start}}+l\frac{\boldsymbol{p}_{\mathbf{end}}\boldsymbol{-}\boldsymbol{p}_{\mathbf{start}}}{\left\Vert\boldsymbol{p}_{\mathbf{end}}\boldsymbol{-}\boldsymbol{p}_{\mathbf{start}} \right\Vert} \right)-\boldsymbol{p}_{\mathbf{tip}}\left( i+1 \right) \right\Vert\right\} (cm)$. |
| Distribution of velocity　(Idle/Low/Middle/High/Very high) | Idle | (%) | The ratio of the number of frames whose instrument velocity belongs to a certain velocity band to the total number of frames. | $\frac{\left\vert n_{\mathrm{Idle}} \right\vert}{n} : n_{\mathrm{Idle}}=\left\{ i\in\left( 0,\ldots n \right) \vert0\leq v\left( i \right)<0.5 \right\} \left( \mathrm{cm}/s \right)$, where $v\left( i \right)$ is the tip velocity of an instrument in frame $i$. |
|  | Low |  |  | $\frac{\left\vert n_{\mathrm{Low}} \right\vert}{n} : n_{\mathrm{Low}}=\left\{ i\in\left( 0,\ldots n \right) \vert0.5\leq v\left( i \right)<2.0 \right\} \left( \mathrm{cm}/s \right)$. |
|  | Middle |  |  | $\frac{\left\vert n_{\mathrm{Middle}} \right\vert}{n} : n_{\mathrm{Middle}}=\left\{ i\in\left( 0,\ldots n \right) \vert2.0\leq v\left( i \right)<5.0 \right\} \left( \mathrm{cm}/s \right)$. |
|  | High |  |  | $\frac{\left\vert n_{\mathrm{High}} \right\vert}{n} : n_{\mathrm{High}}=\left\{ i\in\left( 0,\ldots n \right) \vert5.0\leq v\left( i \right)<12.0 \right\} \left( \mathrm{cm}/s \right)$. |
|  | Very high |  |  | $\frac{\left\vert n_{\mathrm{Veryhigh}} \right\vert}{n} : n_{\mathrm{Veryhigh}}=\left\{ i\in\left( 0,\ldots n \right) \vert5.0\leq v\left( i \right)<12.0 \right\} \left( \mathrm{cm}/s \right)$. |
| Depth path length (DPL) | | (m) | Total length of tip trajectory of an instrument along its sheath axis. | $\sum_{i=1}^{n} \left\vert x_{L\left( i-1 \right)\mathrm{tip}}\left( i \right)-x_{L\left( i-1 \right)\mathrm{tip}}\left( i-1 \right) \right\vert$, where $x_{L\left( i-1 \right)\mathrm{tip}}\left( i \right)$ is an instrument’s tip position on the X-axis in frame $i$, which is represented in the local coordinate system of the instrument in frame $\left( i-1 \right)$. The positional vector of the tip of an instrument $\boldsymbol{v}_{\mathbf{L}\left( \boldsymbol{i-1} \right)\mathbf{tip}}\left( i \right)=\left[ \begin{matrix} x_{L\left( i-1 \right)\mathrm{tip}}\left( i \right) & y_{L\left( i-1 \right)\mathrm{tip}}\left( i \right) & z_{L\left( i-1 \right)\mathrm{tip}}\left( i \right) \end{matrix} \right]$ is represented as follows:  $\boldsymbol{v}_{\mathbf{L}\left( \boldsymbol{i-1} \right)\mathbf{tip}}\left( i \right)=\boldsymbol{M}\left\{ \boldsymbol{v}_{\mathbf{Gtip}}\left( i \right)-\boldsymbol{v}_{\mathbf{Go}}\left( i-1 \right) \right\}$,  where $\boldsymbol{M}$ is **the** rotation matrix, represented as follows: $\boldsymbol{M=}R_{x}\left( -\alpha_{i-1} \right)R_{y}\left( -\beta_{i-1} \right)R_{z}\left( -\gamma_{i-1} \right)$. Here, $R_{x}, R_{y}, and R_{z}$ are rotation matrices around X, Y, and Z-axes respectively, and $\alpha_{i-1} , \beta_{i-1}, and \gamma_{i-1}$ are attitude angles around X, Y, and Z-axes (Roll, Pitch, and Yaw) in frame $\left( i-1 \right)$ respectively. These angles are represented as Z-Y-X euler angles. |
| Depth velocity (DV) | | $\left( \mathrm{cm}/s \right)$ | Average tip velocity of an instrument along the sheath axis. | $\frac{1}{n}\sum_{i=1}^{n} \frac{\left\vert x_{L\left( i-1 \right)\mathrm{tip}}\left( i \right)-x_{L\left( i-1 \right)\mathrm{tip}}\left( i-1 \right) \right\vert}{DT}$ , where $DT$ (s) is the measurement period of the Mocap system. In this study, the measurement frequency was set to 30 Hz, and $DT$ was calculated as $DT=\frac{1}{30}=0.0333\cdots$ (s). |
| Number of opening/closing operations (NOC) | | (times) | Total number of iterations of opening and closing the jaws of forceps. “A series of opening and closing the forceps once” is counted as “one iteration”. |  |
| Average gripper rotation angle (AGRA) | | ($^{\circ}$) | Average of the gripper rotation angle of grasping forceps during the entire task all cases. | $\frac{1}{n}\sum_{i=1}^{n} \theta_{\mathrm{gripper}}\left( i \right)$, where $\theta_{\mathrm{gripper}}\left( i \right)$ is the gripper rotation angle of an instrument in frame $i$*.* |
| Average attitude angle　(Roll/Pitch/Yaw) | Roll | ($^{\circ}$) | Average attitude angle (Roll/Pitch/Yaw) of an instrument entire the task. | $\frac{1}{n}\sum_{i=1}^{n} \alpha\left( i \right)$. |
|  | Pitch |  |  | $\frac{1}{n}\sum_{i=1}^{n} \beta\left( i \right).$ |
|  | Yaw |  |  | $\frac{1}{n}\sum_{i=1}^{n} \gamma\left( i \right).$ |
| Angular length (AL-Roll/AL-PitchYaw) | AL-Roll | ($^{\circ}$) | The total change in the attitude angle of an instrument. This was calculated for two items: the attitude angle around the sheath axis of an instrument (Roll) and that of the inserting point (Pitch and Yaw). | $\sum_{i=1}^{n} \sqrt{\left( \alpha_{i}-\alpha_{i-1} \right)^{2}}$. |
|  | AL-Pitch&Yaw |  |  | $\sum_{i=1}^{n} \sqrt{\left( \beta_{i}-\beta_{i-1} \right)^{2}+\left( \gamma_{i}-\gamma_{i-1} \right)^{2}}$. |
| Working area (WA) | | ($\mathrm{cm}^{2}$) | Moving area of the tip of the instrument in a plane orthogonal to the target object (Y-Z plane in Figure 1). | $\left( y_{\mathrm{tip}\left( q=0.975 \right)}-y_{\mathrm{tip}\left( q=0.275 \right)} \right)\left( z_{\mathrm{tip}\left( q=0.975 \right)}-z_{\mathrm{tip}\left( q=0.275 \right)} \right)$, where $y_{\mathrm{tip}\left( q=0.975 \right)}$ is the 97.5-percentile of an instrument’s tip position in the Y-axis, and $y_{\mathrm{tip}\left( q=0.275 \right)}$ is that of the 2.5-percentile.$z_{\mathrm{tip}\left( q=0.975 \right)}$ and $z_{\mathrm{tip}\left( q=0.275 \right)}$ are calculated in the same way in the Z-axis. |
| Average inserting time (AIT) | | (s) | Average operational time of applying the clip procedure using Hem-o-lok. This index is calculated as the time between inserting Hem-o-lok into the box trainer and its removal. | $\frac{1}{n_{\mathrm{inseting}}}\sum_{i=1}^{n_{\mathrm{inseting}}} T_{\mathrm{inserting}}\left( i \right)$,  where $n_{\mathrm{inseting}}$ is the total number of inserting times of Hem-o-lok, and $T_{\mathrm{inserting}}\left( i \right)$ is the inserting time on the $i$th insertion. |
