# Supplementary material for: Objective evaluation of laparoscopic surgical skills in wet lab training based on motion analysis and machine learning
Source: Langenbecks Arch Surg. 2022 Apr 8;407(5):2123–32. doi: 10.1007/s00423-022-02505-9 (PMC9399206; doi:10.1007/s00423-022-02505-9)
Supplement: Supplementary file 4 — Supplementary file4 (DOCX 917 KB) [file 423_2022_2505_MOESM4_ESM.docx]

Supplementary Table 2. Summary of hypotheses for each item’s estimation derived from the discussion between the two authors
