# Supplementary material for: Objective evaluation of laparoscopic surgical skills in wet lab training based on motion analysis and machine learning
Source: Langenbecks Arch Surg. 2022 Apr 8;407(5):2123–32. doi: 10.1007/s00423-022-02505-9 (PMC9399206; doi:10.1007/s00423-022-02505-9)
Supplement: Supplementary file 5 — Supplementary file5 (DOCX 14 KB) [file 423_2022_2505_MOESM5_ESM.docx]

Supplementary Table 3. Details of the 4 algorithms and candidate parameters for the grid search

| Regression algorithms | Description | Hyperparameters and grid serach range |
| --- | --- | --- |
| Support Vector Regression (SVR) | SVR is a regression method using Support Vector Machine (SVM). In this method, a linear regression line is determined by the support vector that is the closest point of the hyperplane calculated by SVM. However, by Radial Basis kernel Function, (RBF), SVR can be applied to non-linear problems. | Cost parameter C: $C=2^{-5}, 2^{-4}, \ldots, 2^{10}$Band width of dead zone ϵ: $\epsilon=2^{-10}, 2^{-9},\ldots, 2^{0}$Coefficient of RBF $\gamma$: $\gamma=2^{-20}, 2^{-19}, \ldots, 2^{10}$ |
| Principal Component Analysis (PCA)-SVR | This is the combined method of PCA and SVR. In this method, principal component scores of the input data are calculated, and the dimensions of the data are reduced by the cumulative proportion. After the dimensionality reduction, the input data are processed by SVR, and the regression line is calculated. | Threshold of the cumulative proportion $P_{\mathrm{th}}$: $P_{\mathrm{th}}=0.7, 0.8, 0.9$Note that the hyper parameter of SVR ($C, \epsilon, \gamma$) are the same as described above. |
| Ridge Regression (RR) | RR is a linear regression method using the least square (LS) method. In this method, the regulation term is added to the objective function of the LS method, and can be applied to regression problems that have multiple correlations in input data. | The coefficient of the regulation term $\alpha$:  $\alpha=2^{-10}, 2^{-9}, \ldots, 2^{15}.$ |
| Partial Least Squares Regression (PLSR) | PLSR is a linear regression method using the PCA and LS methods. In this method, explanatory and objective variables of data are used as input of PCA, and principal component scores are calculated so that the covariance between the principal component score and objective variable is maximized. The processed data are used as input of the LS method and a regression line is calculated. | The number of input vectors $N_{\mathrm{input}}$:  $N_{\mathrm{input}}=1, 2, \ldots, 5$. |
