# Supplementary material for: Objective evaluation of laparoscopic surgical skills in wet lab training based on motion analysis and machine learning
Source: Langenbecks Arch Surg. 2022 Apr 8;407(5):2123–32. doi: 10.1007/s00423-022-02505-9 (PMC9399206; doi:10.1007/s00423-022-02505-9)
Supplement: Supplementary file 6 — Supplementary file6 (DOCX 64 KB) [file 423_2022_2505_MOESM6_ESM.docx]

Supplementary Table 4. Summary of Spearman’s rank correlation coefficients between GOALS scores assessed by two experts
